# Preclinical Development of a Novel, Orally-Administered Anti-Tumour Necrosis Factor Domain Antibody for the Treatment of Inflammatory Bowel Disease

**DOI:** 10.1038/s41598-018-23277-7

**Published:** 2018-03-21

**Authors:** J. Scott Crowe, Kevin J. Roberts, Timothy M. Carlton, Luana Maggiore, Marion F. Cubitt, Simon Clare, Katherine Harcourt, Jill Reckless, Thomas T. MacDonald, Keith P. Ray, Anna Vossenkämper, Michael R. West

**Affiliations:** 1 VHsquared Ltd., 1 Lower Court, Copley Hill, Cambridge Road, Babraham, Cambridge, CB22 3GN UK; 2 Wellcome Sanger Institute, Wellcome Genome Campus, Hinxton, CB10 1SA UK; 3 RxCelerate Ltd. Babraham Research Campus, Cambridge, CB22 3AT UK; 4 Blizard Institute, Barts and the London School of Medicine, Queen Mary University of London, London, E1 2AT UK

## Abstract

TNFα is an important cytokine in inflammatory bowel disease. V565 is a novel anti-TNFα domain antibody developed for oral administration in IBD patients, derived from a llama domain antibody and engineered to enhance intestinal protease resistance. V565 activity was evaluated in TNFα-TNFα receptor-binding ELISAs as well as TNFα responsive cellular assays and demonstrated neutralisation of both soluble and membrane TNFα with potencies similar to those of adalimumab. Although sensitive to pepsin, V565 retained activity after lengthy incubations with trypsin, chymotrypsin, and pancreatin, as well as mouse small intestinal and human ileal and faecal supernatants. In orally dosed naïve and DSS colitis mice, high V565 concentrations were observed in intestinal contents and faeces and immunostaining revealed V565 localisation in mouse colon tissue. V565 was detected by ELISA in post-dose serum of colitis mice, but not naïve mice, demonstrating penetration of disrupted epithelium. In an *ex vivo* human IBD tissue culture model, V565 inhibition of tissue phosphoprotein levels and production of inflammatory cytokine biomarkers was similar to infliximab, demonstrating efficacy when present at the disease site. Taken together, results of these studies provide confidence that oral V565 dosing will be therapeutic in IBD patients where the mucosal epithelial barrier is compromised.

## Introduction

The cytokine tumour necrosis factor alpha (TNFα) plays a central pathogenic role in inflammatory bowel disease (IBD). Increased levels of TNFα in the lamina propria of the gut mucosa drive chronic inflammatory processes that damage intestinal epithelial cells, resulting in the loss of mucosal barrier integrity and contributing to the breakdown of intestinal immune homeostasis^1,2^. Antibodies with specific TNFα neutralising activities, including infliximab, adalimumab, certolizumab and golimumab, are highly effective for the treatment of IBD^3,4^. However, these biological agents are administered parenterally and consequently are distributed systemically before reaching the gastrointestinal (GI) mucosal tissues. The intravenous or subcutaneous route of administration is inconvenient both for the patient and medical practitioners, particularly for administration inside the hospital setting. There are also major safety concerns, including infusion reactions and increased risk of opportunistic infections associated with systemic suppression of the immune system. Oral administration of an anti-TNFα therapy would ensure delivery direct to intestinal tissues that are affected by TNFα overproduction while limiting systemic exposure and immunosuppression in tissues not involved in the inflammatory disease process.

Multiple studies have provided support for the concept of an oral anti-TNFα for the management of IBD. Orally administered polyclonal avian and bovine anti-TNFα antibodies were effective in rodent colitis models, despite the fact that these antibodies are degraded by gastrointestinal proteases^5,6^. Furthermore, it has been shown that local intestinal delivery of lactobacilli secreting a domain antibody construct that neutralises murine TNFα was able to suppress colonic inflammation in a mouse model of IBD^7^. A recent study testing the Avaxia oral bovine colostral polyclonal anti-TNFα product AVX-470 in patients with ulcerative colitis (UC) showed efficacy trends for clinical, endoscopic and biomarker endpoints^8,9^. However, although AVX-470 was well tolerated, the greatest endoscopic improvements were limited to the proximal colon, possibly reflecting a gradient of active antibody along the axis of the colon^8^ or susceptibility to proteolytic inactivation during transit through the colon. Indeed, recent *in vitro* studies have shown that proteases present in IBD colonic mucosal tissue may contribute to a loss of integrity and TNFα-neutralising activity of conventional antibodies including infliximab and adalimumab^10^. An oral antibody optimised for resistance to proteases present in the luminal contents of the colon as well as those in inflamed tissue would have increased potential for persistence and neutralisation of TNFα in the mucosa.

Llama heavy chain only variable domain antibodies (VHHs) retain the potency and specificity of conventional antibodies, but have unique properties including their small size (12–15 kDa), solubility and intrinsic physicochemical stability^11^, that make them an excellent scaffold for developing an oral therapy. The leakiness of the mucosal epithelium in patients with Crohn’s disease (CD)^12,13^ and the small size of domain antibodies should facilitate penetration of the diseased tissue within the GI tract. Results from the AVX-470 clinical study provide compelling evidence in UC patients that the permeability of the mucosal epithelium to large proteins (up to 150 kDa) is increased^9^. Therefore, a much smaller, protease-resistant, domain antibody should easily access the lamina propria to achieve TNFα neutralisation at the site of production. The aim of this study was to investigate the concept and feasibility of oral dosing with V565 a novel, oral anti-TNFα domain antibody for the treatment of IBD in humans.

## Materials and Methods

### Reagents and Antibodies

V565 and ID-34F (V565 with an aspartate to glutamate amino acid substitution at position one) protease resistant anti-TNFα domain antibodies, ID-25F (homobihead of ID-34F in which the two monomers are joined by a (G_4_S)_6_ linker), and ID-2A (control domain antibody directed against a non-immune target) were produced from *S. cerevisiae* and purified using CaptoS ion exchange chromatography. Q62C9 (an un-engineered anti-TNFα domain antibody, unrelated to V565) was produced with 6xHis and FLAG tags, expressed from *E. coli* and purified using Talon affinity chromatography. Recombinant human sTNFα: Thermo Fisher Scientific, PHC 3013 and PHC 3015; recombinant cynomolgus monkey sTNFα: QVQ, Utrecht, Netherlands. Infliximab, adalimumab, and etanercept were clinical grade from Johnson & Johnson, AbbVie, and Pfizer respectively. Biotinylated adalimumab was a custom preparation from LGC, Fordham, UK.

Antibodies were as follows; biotinylated anti-human TNFα antibody (Peprotech, P31ABt); Rabbit anti-V565 polyclonal antibody (Eurogentec, custom); anti-VHH pAb (BAC, custom); goat anti-rabbit IgG Alexa Fluor 594 antibody (Molecular Probes); Swine anti-rabbit–HRP (Dako P0217); Rabbit anti-human IgG-HRP (Dako P0214); Extravidin-HRP (Sigma E2886).

Cell lines: Mouse L929 cells were purchased from the ECACC (Cat. No. 85011425), HEK-293 TLR2/NFkB/SEAP cells were from Imgenex (Cat. No. IML-102) and CHO Flp-In cells engineered for stable expression of tmTNFα were purchased from Invitrogen.

### V565 *In vitro* pharmacology studies

#### Biosensor Studies

The binding affinities of V565 and adalimumab Fab for human sTNFα were determined at 25 °C, using a switchSENSE DRX 2400 Instrument (Dynamic Biosystems, Munich, Germany^14^). For adalimumab Fab measurements, the sTNFα was captured onto a switchSENSE MPC-48-2-Y1 biochip and antibody capture was measured. However, due to its small size, reliable data could not be obtained for V565 in this orientation. Instead, V565 was coupled to the chip and sTNFα capture was measured.

### ELISA Assays

Unless otherwise stated, antibody and cytokine dilutions were prepared in block buffer (1% BSA in PBS, pH 7.4). Protocols for these ELISAs are given in the Supplementary Methods.

#### V565 Inhibition of Cellular Responses to Soluble and Membrane TNFα

V565 neutralisation of human or cynomolgus monkey TNFα activity was compared with adalimumab in two cell-based assays by measuring either inhibition of sTNFα-induced cytotoxicity of L929 cells or inhibition of sTNFα or tmTNFα activation of NF-κB/SEAP reporter-gene transfected HEK cells. All cell lines were cultured in DMEM (Sigma, D6429), 10% FBS, and Pen/Strep at 37 °C and 5% CO_2_. Geneticin, at 500 µg/mL (G418; Invitrogen, 10131027), was used for HEK-293 cells. Details of experimental conditions are shown in Supplementary Methods.

#### *In vitro* Resistance of V565 to Inactivation by Small Intestinal, Ileal, Faecal and Inflammatory Proteases

Mouse small intestinal (SI) supernatant: Naïve C56BL/6 mice were culled, and the small intestine was ligated, removed, flushed with 1 ml 0.85% saline and the contents homogenised and centrifuged to remove solid material. Supernatants were pooled, aliquoted and frozen at −80 °C until use.

Human ileal supernatant: Ileal contents from six individuals (Seralab, Tissue Solutions) were centrifuged to remove solid material. Equal volumes of each supernatant were pooled, aliquoted and frozen at −80 °C until use.

Human faecal supernatant: A total of 165 g faeces from 10 healthy donors was homogenised in 205 ml 0.85% saline and centrifuged to remove solid material. Supernatants were pooled, aliquoted and frozen at −80 °C until use.

Antibody solutions were prepared at 250 µg/ml in PBS, pH 7.4 0.1% BSA, and used in digestion reactions at 20 µg/ml in relevant matrices. As a 0 hour time point, one aliquot was immediately mixed 1:1 with stop solution (2% BSA, 5 mM EDTA, 2x SigmaFast protease inhibitor cocktail (Sigma S8820), and 1 mM PMSF (Sigma 93482)) and frozen at −80 °C. Further aliquots were incubated at 37 °C until the desired time, whereupon an equal volume of stop solution was added and reactions were frozen. Samples were analysed by TNFR2-TNFα ELISA.

### Disease-associated MMPs

Upon activation, recombinant human MMP3 (513-MP-010) and MMP12 (917-MP-010) (R&D Systems, UK) were co-incubated for 22 h with ID34F, etanercept, or adalimumab using conditions described by Biancheri *et al*.^10^. Reaction products were analysed by Western blotting as described in the Supplementary Methods.

### *In vivo* Studies of GI Stability and Distribution of V565 in the Mouse

The care and use of all mice was in accordance with UK Home Office regulations, UK Animals Scientific Procedures Act 1986 under the project licence PPL80/2596. This licence was reviewed and approved by The Wellcome Trust Sanger Institute Animal Welfare and Ethical Review Committee.

The mice were given food (LabDiet 50213) and water ad labitum. Mice were house in an IVC cage system with aspen wood chip, card tunnel and nesting material as environmental enrichment. All procedures were carried out during the light cycle.

#### GI Transit in Mice Following Oral Administration

C57BL/6n male mice were pre-dosed by oral gavage with 0.1 ml of a gastro-protective vehicle (0.1 M NaHCO_3_ containing 400 mg/ml Marvel milk), then dosed after 15 minutes with 0.2 ml vehicle, or vehicle containing 140 µg V565. Mice were culled at 3 h or 7 h and luminal contents of the stomach, small intestine, caecum and colon were recovered. Faecal pellets were collected at hourly intervals (1 h to 7 h) after dosing. Gastrointestinal luminal contents and faecal samples were weighed, homogenised in 4 volumes extraction buffer (0.6 M NaCl, 1% BSA, 0.05% Tween 20, 2x SigmaFast protease inhibitor cocktail in PBS) and centrifuged at 13000 rpm at 10 °C for 20 mins. The supernatants were stored at −80 °C until analyses.

#### V565 Mucosal and Sub-Mucosal Tissue Penetration in DSS-Colitis Mice

Twelve C57BL/6n male mice were used: three control mice and nine mice where colitis was chemically induced by the administration of 2% dextran sodium sulphate (DSS)^15^. All mice were initially dosed with 0.1 ml vehicle by oral gavage. 15 minutes later, both naïve and DSS-treated mice were fed 0.2 ml of either 700 µg/ml V565 in vehicle or vehicle only. Two DSS colitis mice were sacrificed at 1, 3, 5, 7, 9 and 14 h post-dosing. Naïve mice were sacrificed at 3 h. Colons were removed from the sacrificed mice, cut into three segments, and contents removed. Colons were embedded in OCT and frozen in liquid nitrogen for cryo-sectioning and immunocytochemistry. Colon contents were analysed for V565 concentration using the TNFα binding ELISA.

#### Immunocytochemical Analysis of V565 in Mouse Colon Tissue

Colon sections were fixed in ice cold acetone, dried, blocked, and incubated with 10 µg/ml of rabbit polyclonal IgG, or rabbit anti-V565 pAb 1952 overnight at 4 °C. After washing, sections were incubated with 20 µg/ml anti-rabbit Alexa 594 and 1 µg/ml Hoechst 33342 for 6 h, washed, air dried and mounted under a coverslip in Citifluor, AFI. Sections were examined using an Olympus AX70 microscope and images were captured with Image Pro-Plus (v7.0, Media Cybernetics) using fixed exposures.

#### Human IBD Tissue *ex vivo* Biopsy Studies

Research ethics committee approval (reference 10/H0704/73) for studies using human tissue was obtained from the NRES Committee London – City & East. The study was also approved by the local Barts and The London School of Medicine and Dentistry QMUL Joint R&D office. All aspects of the work described have been done following Good Clinical Practice and Good Clinical Laboratory Practice guidelines. All patients took part in the study after giving informed written consent.

Biopsy tissue was obtained from inflamed colonic mucosa during routine endoscopy of patients with CD or UC. *Ex vivo* IBD biopsy cultures for the analysis of TNFα-dependent phosphoproteins and cytokine biomarkers were run as described by Vossenkämper *et al*.^16^. CD or UC biopsies were incubated in organ culture for 24 h with the addition of the antibodies, either ID-2A (unrelated control domain antibody), V565, IgG1 (unrelated control mAb) or infliximab (clinical control mAb) at the concentrations shown. Supernatants and tissue samples collected at the end of the experiment were snap-frozen and stored at −70 °C.

For analysis of phosphoproteins, tissue samples were thawed, lysed in RIPA Buffer (Sigma-Aldrich, R0278) supplemented with phosphatase inhibitor cocktail 2 (Sigma-Aldrich) and protease inhibitor cocktail (Sigma-Aldrich, P8340) and the lysate supernatants diluted to 1 mg/ml protein. The phosphorylation status of 150 µg lysate proteins was determined using PathScan RTK signalling arrays (Cell Signalling Technology, Danvers, MA). Chemiluminescent signals of all arrays were detected on X-ray films, and the pixel intensities were measured using ImageJ software. Biopsies from four CD or UC patients were tested for each antibody treatment and the array signals used to calculate mean intensity values for each of the 39 phosphoproteins in the treatment groups, as well as total intensity values for all 39 phosphoproteins in the biopsies from each treatment group.

For the measurement of cytokines, the frozen 24 h culture supernatants were thawed and analysed for levels of IL-1β, IL-6 IL-8, IL-17A, TNFα and IL-10 using Luminex multiplexed cytokine assay kits (R&D Systems) and an R&D Systems MAGPIX® analyser. Mean values ± SDs were calculated for the levels of spontaneous cytokine production measured in biopsy culture supernatants from each treatment group.

### Data Availability

All data generated or analysed during this study are included in this published article (and its Supplementary Information files).

## Results

VHH domain antibodies with potent TNFα-neutralising activity were isolated from a llama VHH phage display library and a lead VHH with some intrinsic resistance to intestinal proteases was selected. Structural engineering of the lead molecule was undertaken to enhance resistance of the VHH to inactivation by intestinal proteases, while retaining potent neutralising activity against human and cynomolgus monkey TNFα. The fully optimised Vorabody™, V565, is 12.6 kDa, 115 amino acids in length and is manufactured in *Saccharomyces cerevisiae*.

### *In Vitro* Pharmacology

#### V565 Neutralises Soluble and Transmembrane Human TNFα With Potency Comparable to Adalimumab

V565 was tested for potency alongside the clinical comparator antibody adalimumab. SwitchSENSE biosensor technology, which examines biophysical binding properties, demonstrated that an adalimumab Fab fragment and V565 both bound to human sTNFα with picomolar affinity (Table 1). Furthermore, V565 fully neutralised the binding of sTNFα to both TNFR1 and TNFR2 receptors with a similar potency to that of adalimumab in an ELISA format (Table 1).

**Table 1:** V565 displays similar potency to approved anti-TNFα agents.

Assay Method	Antibody	TNFα	Functional Readout	Potency IC_50_ or K_D_ (nM)
Biosensor	V565	sTNFα	TNFα association and dissociation kinetics	0.017^b^
Biosensor	adalimumab^a^	sTNFα	TNFα association and dissociation kinetics	0.005^b^
ELISA	V565	sTNFα	Inhibition of TNFα-TNFR2 interaction	0.41^c^
ELISA	adalimumab	sTNFα	Inhibition of TNFα-TNFR2 interaction	0.21^c^
ELISA	V565	sTNFα	Inhibition of TNFα-TNFR1 interaction	0.6^c^
ELISA	adalimumab	sTNFα	Inhibition of TNFα-TNFR1 interaction	0.2^c^
L929 cell viability	V565	sTNFα	Inhibition of TNFα-induced cell cytotoxicity	0.27^d^
L929 cell viability	adalimumab	sTNFα	Inhibition of TNFα-induced cell cytotoxicity	0.17^d^
HEK-NFkB-cells	V565	sTNFα	Inhibition of TNFα-induced NFkB-SEAP	0.3^d^
HEK-NFkB-cells	V565	tmTNFα	Inhibition of tmTNFα-CHO induced NFkB-SEAP	5.2^d^
HEK-NFkB-cells	adalimumab	tmTNFα	Inhibition of mTNFα-CHO induced NFkB-SEAP	3.1^d^
HEK-NFkB-cells	infliximab	tmTNFα	Inhibition of mTNFα-CHO induced NFkB-SEAP	3.8^d^

^a^Adalimumab Fab fragment.

^b^K_D_ equilibrium dissociation constant.

^c^IC_50_ concentration of V565 or adalimumab required to achieve 50% inhibition of maximal sTNFα-binding to TNFR1 or TNFR2 in a plate ELISA format.

^d^IC_50_ concentration of V565 or adalimumab required to achieve 50% inhibition of maximal TNFα-induced response.

TNFα-neutralising activity was also demonstrated in various cellular assays. V565 potently neutralized the cytotoxic activity of sTNFα on L929 cells, with complete restoration of L929 cell viability at maximal V565 concentrations. In HEK-293-NF-κB/SEAP reporter cell cultures, V565 potently inhibited alkaline phosphatase production as a result of stimulation by either sTNFα or human transmembrane-TNFα (tmTNFα) expressed on the surface of CHO cells in a co-culture system (Table 1). In general, the potencies of V565 in these assays were found to be two to three-fold less in ELISA assays and only ≈1.7-fold less in cellular assays compared with the bivalent adalimumab.

#### V565 Specificity and Cross-Reactivity With Other Species TNFαs

The specificity of V565 was tested against other human proteins including lymphotoxin alpha (hLTA), the most closely-related human protein sequence in the NCBI BLAST database, and human IL-6. No evidence of V565 binding to hLTA or IL-6 was detected by ELISA (Table 2), indicating that off-target binding in humans would be unlikely. The binding of V565 to TNFα from non-human species was also examined. V565 inhibited binding of both soluble human and cynomolgus monkey TNFα to human TNFR2 with similar potency in a competition ELISA, but did not show any binding activity towards rabbit or murine TNFα. In the L929 cellular assay, V565 was a potent and effective inhibitor of the cytotoxicity induced by cynomolgus monkey TNFα, to a similar degree to that of adalimumab (Table 2).

**Table 2:** V565 specifically neutralizes human and cynomolgus monkey TNFα.

Assay	Ligand	Antibody	Functional Readout	Binding/Potency
ELISA^a^	Human TNFα	V565	Inhibition of V565-human TNFα interaction	+++
ELISA^a^	Cyno TNFα	V565	Inhibition of V565-human TNFα interaction	+++
ELISA^a^	Rabbit TNFα	V565	Inhibition of V565-human TNFα interaction	—
ELISA^a^	Murine TNFα	V565	Inhibition of V565-human TNFα interaction	—
ELISA^a^	Human LTα	V565	Inhibition of V565-human TNFα interaction	—
L929 cells	Human TNFα	V565	Inhibition of TNFα-induced cell cytotoxicity	0.27 nM^b^
L929 cells	Cyno TNFα	V565	Inhibition of TNFα-induced cell cytotoxicity	0.08 nM^b^
L929 cells	Cyno TNFα	adalimumab	Inhibition of TNFα-induced cell cytotoxicity	0.05 nM^b^

^a^The ability of human TNFα and TNFα from other species to bind V565 was determined using an adalimumab-TNFα binding competition ELISA format as described in the methods section.

^b^IC_50_ concentration of V565 or adalimumab required to achieve 50% inhibition of maximal TNFα-induced response.

#### V565 is Extremely Resistant to Degradation by GI Proteases

**Figure 1:**
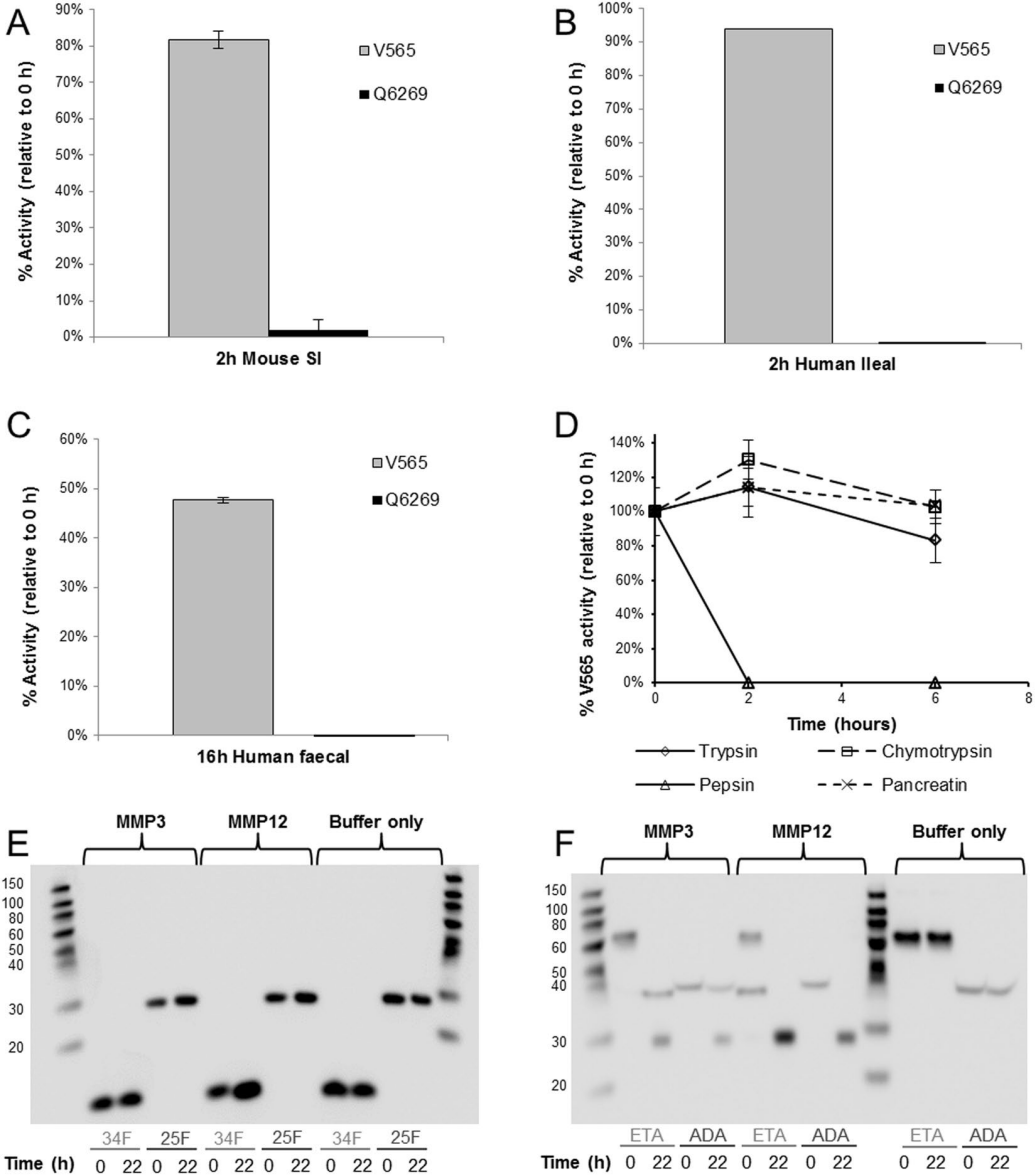
Resistance of V565 and related VHHs to degradation by gastrointestinal proteases. V565 and Q62C9 were incubated in pooled mouse small intestinal supernatant (**A**) or human ileal fluid (**B**) for 2 h, and human faecal extract for 16 h (**C**) at 37 °C. V565 was incubated in 1 mg/ml solutions of purified enzymes over 6 hours (**D**). The remaining TNFα-binding activity in each sample was measured by TNFα-TNFR2 ELISA, and calculated as a percentage against a 0 hour time point. ID34F, ID25F, etanercept (ETA), and adalimumab (ADA) were incubated with recombinant human matrix metalloproteinases (MMPs) for 22 hours. Pre- and post-digestion samples were analysed by Western blotting (**E,F**).

To demonstrate stability in different regions of the GI tract, V565 and another potent anti-TNFα VHH identified during library screening, Q62C9, were incubated in the presence of supernatants prepared from pooled luminal contents of the mouse small intestine and human ileum, and from human faeces. Incubation times were selected to reflect the periods of exposure that might occur during passage through the different regions of the GI tract in humans. The anti-TNFα activity remaining in the final incubation samples was measured by TNFα-TNFR2 ELISA, and showed very high recoveries of V565 in all three digestive matrices. By comparison, Q62C9 was heavily degraded in all three supernatants, confirming their strong proteolytic activity. Only 2% of Q62C9 activity was retained after incubation for 2 hours in mouse SI supernatant, compared with 82% for V565 (Fig. 1A). Furthermore, Q62C9 activity was below the limit of quantification after incubation in either human digestive matrix, compared with 94% of V565 activity after 2 hours in human ileal fluid, and 48% of V565 activity after 16 hours in human faecal material (Fig. 1B,C). V565 was also incubated in purified digestive enzymes in optimal digestion conditions at 37 °C. This revealed that, while sensitive to pepsin, V565 was almost entirely resistant to 1 mg/ml solutions of trypsin, chymotrypsin, and pancreatin over 6 hours (Fig. 1D).

Activated matrix metalloproteinases, including MMP3 and MMP12 that are increased in the inflamed mucosa of patients with IBD, have a wide range of substrates including the anti-TNFα biologics infliximab, adalimumab and etanercept^10^. To investigate resistance to these inflammatory proteases, etanercept and a close analogue of V565 (ID34F) were incubated for 22 h with either human MMP3 or MMP12 and the final products were analysed by Western blotting. While both MMP3 and MMP12 caused degradation of etanercept (Fig. 1E), neither of the proteases caused a significant loss or degradation of ID34F (Fig. 1F).

#### Passage of V565 through the Mouse GI Tract After Oral Dosing

**Figure 2:**
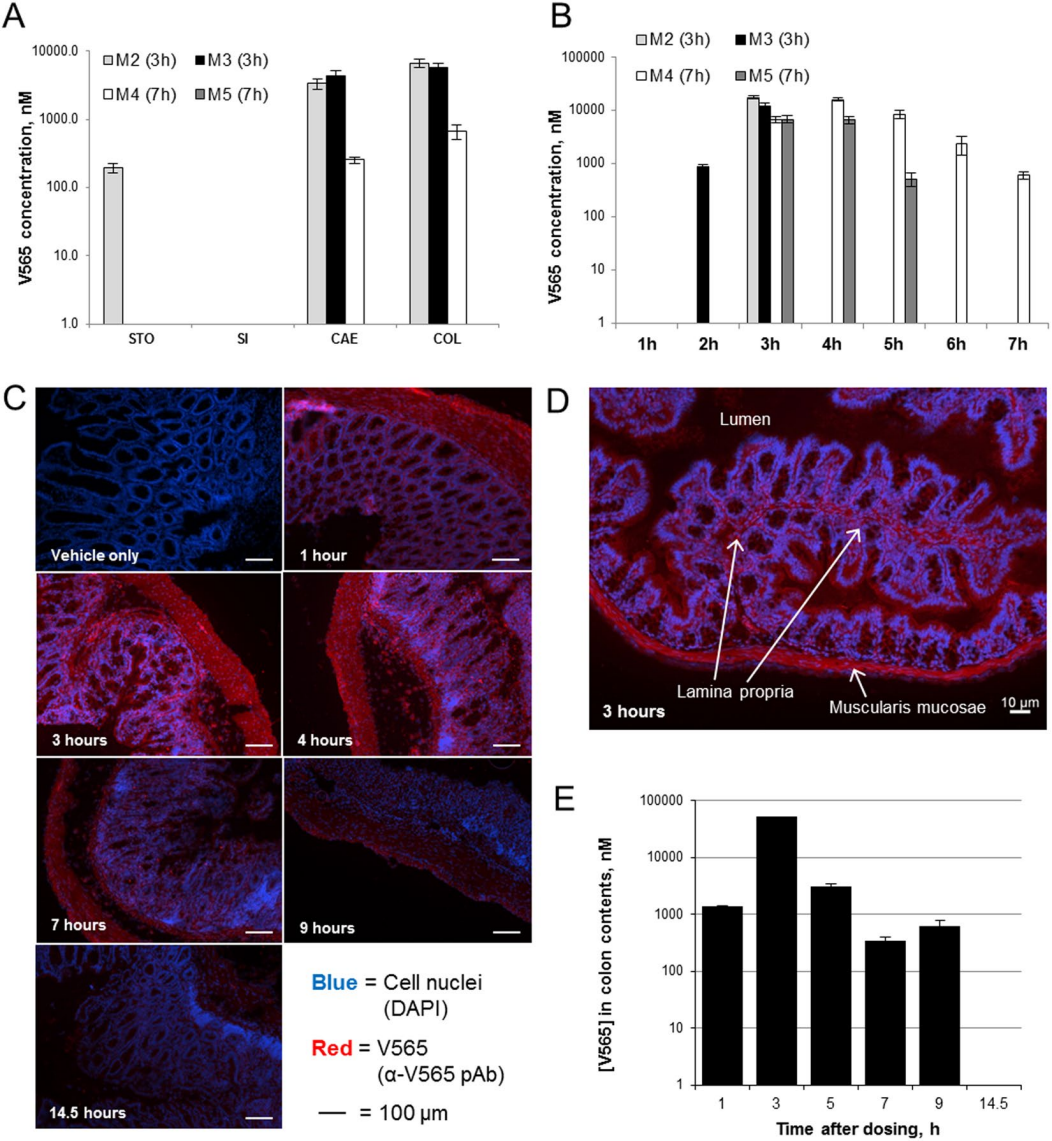
Distribution of V565 in mice following oral administration. Naïve mice were administered 140 µg V565 by oral gavage. Mice were sacrificed after 3 h and 7 h. Concentrations of V565 in the luminal contents of the stomach (STO), small intestine (SI), caecum (CAE) and colon (COL) (**A**) and the faeces (collected hourly until sacrifice) (**B**) were measured by TNFα-biotinylated adalimumab ELISA. Error bars = +/−SD. V565 penetration into the colon mucosa and sub-mucosa of DSS-treated mice after oral gavage of 140 µg was assessed by immunohistochemistry (**C**,**D**) and V565 concentrations in the colon contents at the time of sacrifice were measured by TNFα-biotinylated adalimumab ELISA (**E**). Error bars = +/−SD.

To assess stability *in vivo* during gastrointestinal transit, mice were administered 140 µg V565 in a gastro-protective vehicle by oral gavage. Faeces were collected hourly up to either 3 or 7 hours, when the luminal contents of the stomach, small intestine, caecum and colon were recovered and analysed for V565 concentrations. The transit of V565 through the GI tract was rapid and by 3 hours levels in the stomach and small intestine were already low or undetectable. Meanwhile, the highest concentrations were present in both the caecum and colon (Fig. 2A). V565 levels in faeces excreted during the study varied between animals, but peak concentrations were detected 3-5 hours after dosing in all mice (Fig. 2B). Overall, V565 was shown to transit well through the mouse GI tract delivering high concentrations within the lower GI tract and faeces up to 7 h post-dosing.

#### Penetration of V565 into Colonic Mucosal and Sub-mucosal Tissue in DSS-colitis Mice

For V565 to be effective in patients with CD and UC it must access the lamina propria during passage through the GI tract to neutralise the activity of TNFα at its site of production. To investigate this, V565 was administered to both naïve mice and mice with DSS colitis. Colons were removed at intervals between 1 and 14 hours such that the contents could be analysed and the tissue processed for immunostaining. In naïve mice, very little penetration of V565 into naïve colonic mucosal tissue was observed. In contrast, V565 staining was detected throughout the mucosa, sub-mucosa, and lamina propria of the colon of mice with DSS colitis (Fig. 2C and D). Isotype controls for naïve and DSS tissue showed minimal fluorescence, comparable to or lower than vehicle only controls (data not shown). The staining of V565 in inflamed colon tissue was maximal at 3–5 h, corresponding to the peak concentrations of V565 in the lumen of the colon (Fig. 2E) but had decreased by 7 to 9 hours after dosing, which was consistent with murine intestinal transit times.

#### Serum V565 Levels Following Oral Dosing of Naïve and DSS Colitis Mice

**Figure 3:**
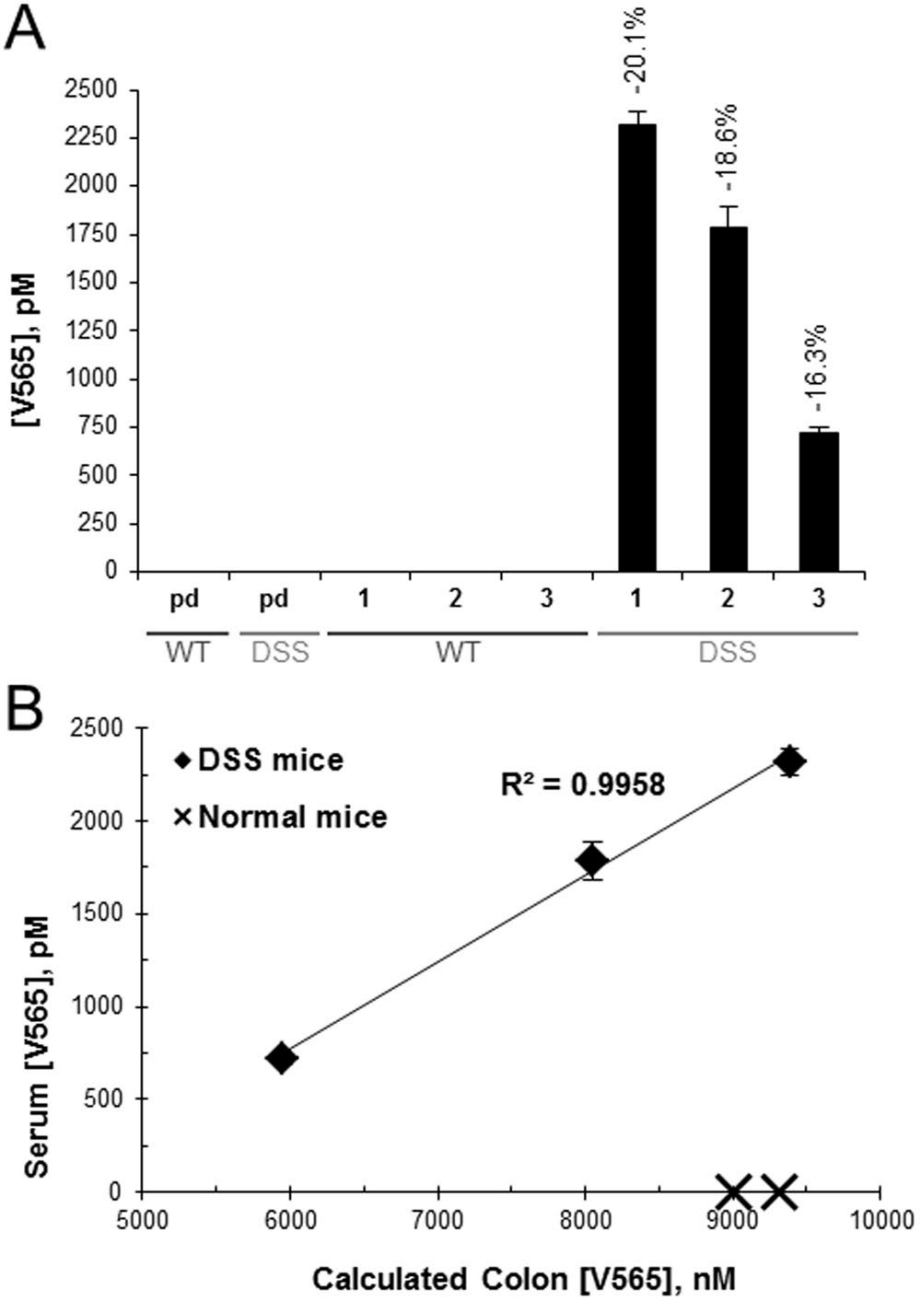
V565 detection in mouse serum after oral dosing. 140 µg V565 was dosed orally to naïve mice (WT) and mice with DSS-induced colitis (DSS). Pre-dose (pd) serum for each group was pooled. Additional serum and colon contents samples were taken 3 hours post-dose and V565 in all samples was quantified by TNFα-binding ELISA. Serum concentrations of V565 with percentage weight loss for each DSS colitis mouse is given above each bar to indicate disease severity (**A**). Error bars = +/−SD. Serum concentrations were co-plotted with V565 concentrations in colon contents (**B**).

Further evidence that V565 was able to penetrate into the submucosa when the colonic epithelial barrier is compromised was provided by analyses of the sera from naïve and DSS colitis mice after oral V565 dosing. As shown in Fig. 3A, the pre-dose sera from both naïve and DSS colitis mice had no detectable V565. This was also the case for the post-dose sera from the naïve mice, despite high colon V565 concentrations (Fig. 3B). However, V565 was detected in sera from all three mice with DSS colitis, with concentrations increasing with disease severity, as judged by % weight loss. The serum concentrations of V565 also correlated with the luminal V565 concentrations in the colons of the colitic mice, where the epithelial barrier was compromised (Fig. 3B).

#### V565 Inhibits Endogenous Protein Phosphorylation in Human IBD Tissue

**Figure 4:**
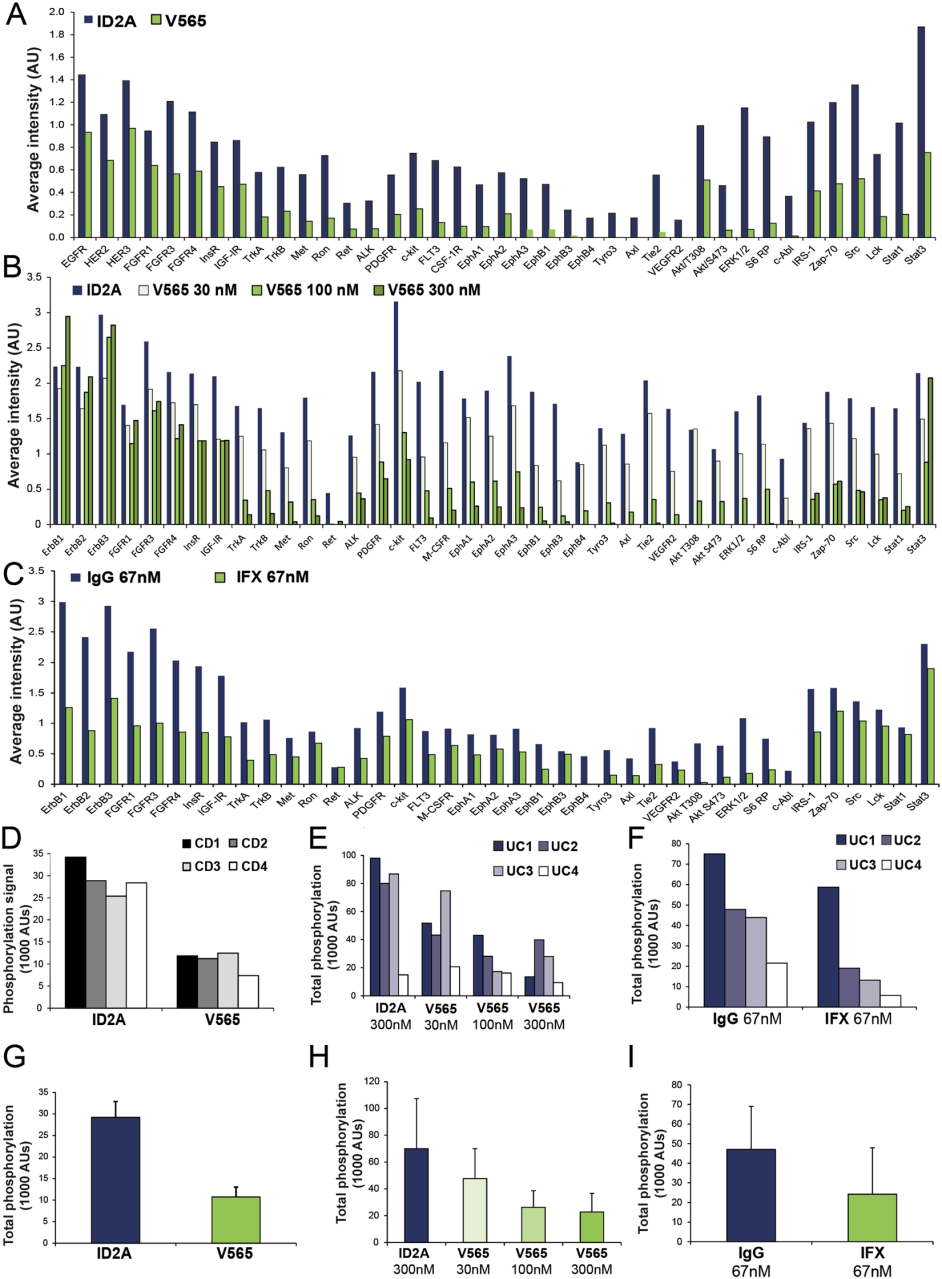
V565 inhibits endogenous phosphorylation of RTKs and signalling proteins in *ex vivo* cultures of inflamed CD and UC tissue. Freshly isolated IBD mucosal biopsies were incubated for 24 h with V565, infliximab (IFX), or corresponding matched control antibodies (ID2A, IgG1). Lysates were analysed on PathScan RTK signalling antibody arrays with chemiluminescent detection, image capture on film and quantification of spot intensities using array analysis software. Average signal intensities (n = 4 patients) obtained for all 39 phosphoproteins were calculated for CD biopsies (CD1-CD4) treated with either ID2A (500 nM) or V565 (250 nM) (**A**), UC biopsies treated with either ID2A (300 nM) or V565 (30 nM, 100 nM or 300 nM) (**B**) or biopsies from the same UC patients treated with mouse IgG1 (67 nM) or IFX (67 nM) (**C**). Total phospho-intensity values calculated from all of the 39 spot intensities on the arrays are shown for the individual biopsies from each patient CD1-CD4 (**D**) and UC1-UC4 (**E**,**F**) with the different treatments grouped together. The corresponding averaged total phospho-intensity values for the CD (n = 4; ±SD) and UC (n = 4; ±SD) treatment groups were calculated also (**G**–**I**).

TNFα is a primary pro-inflammatory cytokine that acts on many cell types and cytokine cascades in diseased tissue, involving multiple receptor signalling pathways and the phosphorylation of receptors, protein kinases and transcription factors. The TNFα-neutralising activities of V565 and infliximab were investigated in *ex vivo* cultures of inflamed IBD colonic tissue, a model that reproduces the IBD tissue environment^16^. Following 24 hours’ incubation with V565 (or infliximab) to neutralise endogenous TNFα, downstream effects on the phosphorylation of RTKs and signalling molecules were assessed using a phosphorylation-state specific antibody array. Signal intensity data collected for the 39 proteins on individual arrays are presented as heat maps (Supplementary Figures S1–S3). The average values per treatment group are shown in Fig. 4A–C.

The majority of proteins in inflamed CD and UC biopsy tissue incubated with the control antibodies (ID2A, IgG) showed prominent phosphorylation signals consistent with the persistence of inflammatory activity in *ex vivo* cultures. Incubation of IBD tissue with V565 (Fig. 4A and B) or infliximab (Fig. 4C) strongly inhibited the phosphorylation of many proteins on the array, with some inhibited by >70% when compared with the controls. Of these, M-CSFR, Tyro-3, Axl, Akt, cAbl, Lck, and Stat1 are known to have functions involved in the regulation of immune cells that contribute to disease pathology, while others have roles in the regulation of angiogenesis (VEGFR2, Tie2) and epithelial cell functions (Ephrin family receptors, Met) or have signalling roles in most cell types (Erk1/2, S6-ribosomal protein) (Supplementary Table S1). Decreased phosphorylation of these proteins is indicative of decreased cell activation and is consistent with V565 neutralisation of endogenous TNFα activity. The similar patterns of phosphoprotein inhibition detected for V565 and infliximab are consistent with the common mechanism of action of these antibodies. When total phospho-intensity values were calculated per patient for all 39 proteins in the control, V565 and infliximab treated biopsies, the inhibitory effects of anti-TNFα treatment observed for individual CD and UC patients is compelling (Fig. 4D–F). Comparison of the total phosphorylation intensity levels measured in the control and V565 treated biopsies from four UC patients (Fig. 4E) showed that tissue phosphorylation was inhibited in the V565 treated cultures with a clear trend for greater inhibition at higher V565 concentrations, reaching 68% inhibition at 300 nM V565 relative to the control VHH. Infliximab inhibited total phosphorylation in biopsies from the same set of UC patients (Fig. 4F) by 49%, relative to the matched IgG isotype control, at a concentration that has been associated with mucosal healing in patients with IBD (10 µg/ml; 67 nM)^17^. The corresponding averaged total phospho-intensity values for each treatment group are also shown (Fig. 4G–I), illustrating the similar trends observed for V565 and infliximab across both sets of IBD patients.

#### V565 Inhibits Spontaneous Cytokine Production by Human IBD Tissue

**Figure 5:**
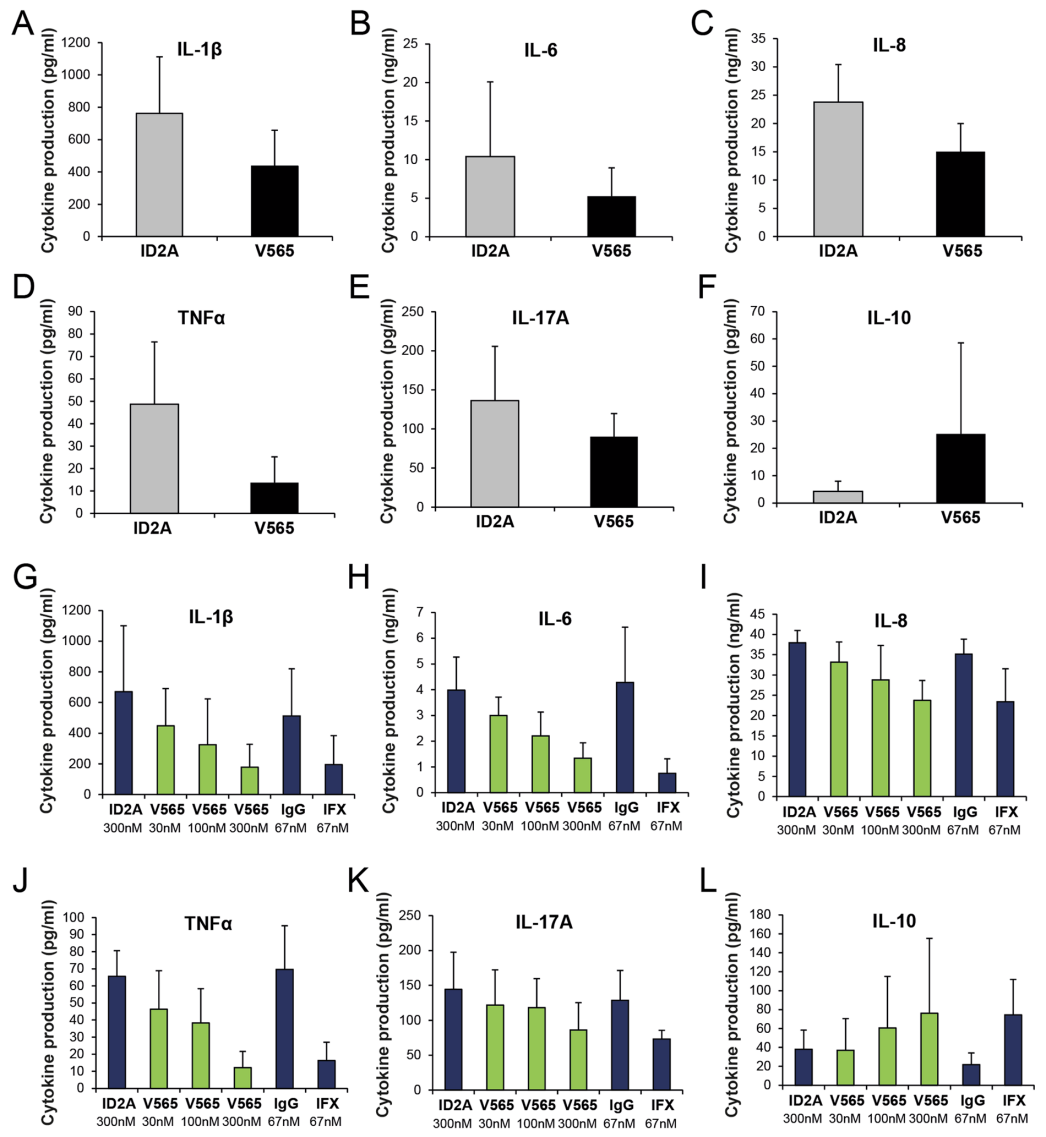
V565 inhibits the spontaneous release of inflammatory cytokines and chemokines in e*x vivo* cultures of inflamed CD and UC tissue. Freshly isolated IBD mucosal biopsies were incubated for 24 h with V565, infliximab (IFX), or corresponding matched control antibodies (ID2A, IgG1). Luminex technology was used to measure simultaneously the levels of IL-1β, IL-6, IL-8, IL-10, TNFα and IL-17A in the supernatants of CD biopsies treated with either ID2A (500 nM) or V565 (250 nM) (n = 6; Mean ± SD) (**A**–**F**), and UC biopsies treated with either ID2A (300 nM), V565 (30 nM, 100 nM or 300 nM), mouse IgG1 (67 nM), or IFX (67 nM) (n = 4; Mean ± SD) (**G**–**L**).

It has been reported previously that in *ex vivo* cultures of inflamed IBD mucosal tissue the spontaneous production of pro-inflammatory cytokines and chemokines is increased^16^. We investigated whether the TNFα-neutralising activity of V565 would have an inhibitory effect on the release of cytokines into culture supernatants recovered from the 24 h CD and UC biopsy cultures described above. In the CD biopsy cultures, V565 inhibited the production of inflammatory cytokines and chemokines with consistent reductions in IL-1β, IL-6, IL-8, TNFα and IL-17A relative to the ID2A controls (Fig. 5A–E). In contrast, the levels of IL-10 in the cultures increased after V565 treatment (Fig. 5F). Treatment of inflamed UC tissue with V565 also inhibited the spontaneous production of the same inflammatory cytokines with maximal inhibitory effects similar to those achieved with the clinical positive control antibody infliximab (Fig. 5G,K). Similarly to the CD tissue, release of IL-10 was consistently increased in the cultures treated with V565 and infliximab (Fig. 5J). However, the inhibitory effects of V565 (and infliximab) on the production of different regulatory cytokines are entirely consistent with the broad downregulation of phosphoproteins noted above.

## Discussion

In the majority of patients with CD, symptoms are limited to the gastrointestinal tract, where the production of TNFα is localised to cells present within mucosal and sub-mucosal tissues. This drives inflammation within the gut wall and the recruitment of additional inflammatory cells that are responsible for the chronicity of the disease and the development of immunopathology^4^. The ability to deliver an oral anti-TNFα therapy, equivalent in potency to current clinical antibody products, avoids the inconvenience of injections or infusions as well as the local reactions and pain associated with these administrations. Orally administered V565 could offer significant improvements in safety due to reduced suppression of the systemic immune response. Local exposure and the potential for oral tolerance^18^ induced against such a therapy may limit the generation of anti-drug antibodies which can shorten the period of efficacy observed with parenterally administered mAbs.

Here, we have shown V565 to be a highly potent inhibitor of not only soluble TNFα, but also tmTNFα, the binding of the latter seeming key for efficacy in CD treatment using full-length antibodies^2,19,20^. In this study, V565 and adalimumab were similarly potent in a tmTNFα reporter cell contact assay but, as reported previously by Shealy *et al*.^21^, both were less potent in this assay compared to the inhibition of soluble TNFα-mediated responses. V565 also neutralises TNFα from the cynomolgus monkey, making it a suitable species for preclinical pharmacokinetic and toxicology studies, similar to other clinical anti-TNFα antibodies. However, oral delivery of effective biologics to disease sites in the human GI tract is not straightforward.

VHHs are inherently more stable physicochemically than conventional antibodies^11^ but are not universally resistant to proteolytic degradation at levels required of an oral biologic^22,23^. Consequently, V565 was specifically selected and engineered for protease resistance, and is considerably more resistant than un-engineered anti-TNFα domain antibodies (Fig. 1A–C). Domain antibodies against other target antigens have been reported to be resistant to proteolytic degradation^22–24^. However, while these studies used 0.001–0.1 mg/ml digestive enzymes for 1 hour, V565 was almost entirely resistant to degradation after 6 hours in 1 mg/mL of trypsin, chymotrypsin, and pancreatin (Fig. 1D), representing a step-change in the protease resistance of biologics.

Although V565 was degraded within 2 hours in an acidic solution of pepsin, for clinical use a formulation using enterically coated mini-tablets to protect V565 during passage through the stomach will provide controlled release in the lower small intestine to target ileo-caecal CD and beyond. V565 survived in human ileal and faecal supernatants and in the presence of inflammatory proteases over time periods that are both stringent and highly relevant for simulating oral delivery in man (Fig. 1B,C,F). High concentrations of V565 were also recovered in the faeces of mice following oral dosing. Taken together, this data provides strong evidence that V565 is highly resistant to mammalian and microbiota-derived intestinal proteases and should successfully survive in the GI tract in man.

To be effective in patients with IBD, V565 will need to penetrate the mucosal epithelial barrier to neutralize TNFα at the site of production in the lamina propria. The barrier function of intestinal epithelial cells is markedly impaired in active IBD, resulting in increased permeability to luminal proteins. *In vitro* studies have shown that in inflamed CD mucosal tissue, proteins including HRP (40 kDa) and ovalbumin (45 kDa) can cross the diseased mucosal epithelium^25,26^. More recently, results of two clinical studies have provided evidence that conventional antibodies can distribute from the gut lumen into the inflamed mucosa in patients with IBD. Endoscopic administration of fluorescent adalimumab to patients with CD led to rapid binding to tmTNFα on the surface of macrophages and CD4+ T cells in the lamina propria^27^. Moreover, AVX-470 bovine IgG antibodies, permeated the gut mucosa of UC patients following oral dosing and, encouragingly, efficacy trends for clinical, endoscopic, and biomarker endpoints were observed at the AVX-470 3.5 g/day dose^8,9^. This strongly supports the hypothesis that orally administered anti-TNFα therapy engages with TNFα in the inflamed gut mucosa.

V565 penetrated into the lamina propria and serum of DSS colitis mice, but not in naïve mice, after oral administration. This is also expected to be the case in humans where the gut lining is intact, further limiting systemic exposure and its associated side effects. The maximum V565 concentration in the mouse lamina propria was achieved at 3 hours and persisted to 7 hours. As V565 does not bind to mouse TNFα, we expect that V565 is rapidly cleared by lymphatic drainage in the mouse but will be retained for longer periods when able to bind to its target antigen in humans. The degree of epithelial damage and histopathological features of murine DSS-colitis reflect those seen in human IBD^28^ and therefore we are confident that V565 will transit into the lamina propria at sites of lesions in IBD patients. Any V565 reaching the serum in man should be rapidly cleared via the kidneys due to its low molecular weight, further limiting systemic exposure.

The lack of cross-species reactivity of V565 with murine TNFα precluded an evaluation of the efficacy of V565 in a mouse model of colitis. Using an alternative, and perhaps more relevant, model involving the treatment *ex vivo* of freshly isolated mucosal tissue from patients with CD or UC, we demonstrated the effectiveness of V565 based on the suppression of protein phosphorylation and inhibition of cytokine production as biomarkers of the underlying inflammatory disease processes. Due to the small number of patient biopsies investigated in this study it was not possible to demonstrate that differences between V565 or infliximab and their control treatment groups were statistically significant using a Wilcoxon sign rank test. Nonetheless, the patterns of inhibition of these markers that resulted from V565 treatment demonstrate that endogenous TNFα production maintains the activation of several cell types including T cells, macrophages, epithelial cells and endothelial cells involved in the disease processes of IBD and are consistent with the broad anti-inflammatory activities of current anti-TNFα therapies. Furthermore, we have observed that the effects of V565 (30 nM to 100 nM) in this model are similar to those produced by 67 nM (10 µg/ml) infliximab, a concentration associated with serum trough levels of the antibody that are required for mucosal healing in patients with IBD^17^. Evidence that V565 inhibited biomarkers of inflammation in biopsies taken from patients with CD and UC is consistent with the efficacy of existing systemically administered anti-TNFα antibodies in both diseases. The two main forms of IBD differ in the location and distribution of intestinal inflammation; CD is a segmental, transmural disorder that can affect the whole gastrointestinal tract while UC is characterized by continuous inflammation of the colon. Although V565 has been developed initially for the treatment CD, the delivery of this antibody directly to ileum and colon may prove to be equally effective in patients with UC where the inflammation is confined to the colonic mucosal and submucosal tissues.

Currently there are no oral anti-TNFα biologics approved for the treatment of IBD; however, several novel agents have been reported and are at various stages of clinical development. AG014 is a strain of genetically modified *Lactococcus lactis* bacteria that was developed for oral administration and designed to express and deliver the anti-TNFα antibody fragment of certolizumab into the intestinal tract^7^. However, following a Phase 1 study, this agent has not been developed further and questions remain regarding the concentration variability and resistance of the antibody fragment to intestinal proteases in patients. More recently, AVX-470 an orally administered bovine polyclonal anti-TNFα antibody was evaluated in patients with active UC^8,9^. In this study, trends towards higher clinical response rates were noted with the greatest improvements in the highest 3.5 g/day AVX-470 treatment group. Additional studies will be needed to evaluate the effects of higher doses and to optimise exposure during transit of the antibody through the colon. In this regard the purification and formulation of a therapeutic product derived from immunised animals, with consistent TNFα-neutralising potency and stability characteristics may prove challenging. The immunomodulatory activity of a plant cell-expressed anti-TNFα fusion protein PRX-106 (Etanercept) is also being investigated for the treatment of IBD. PRX-106 was safe and well tolerated in a phase 1 clinical trial^29,30^. However, unlike clinical anti-TNFα antibodies, systemically administered Etanercept is not an effective treatment in patients with CD or UC^31^. Furthermore, the resistance of oral PRX-106 to digestion by pepsin in the stomach or by inflammatory proteases in disease tissue^10^ is uncertain, so the effectiveness of this material for the treatment of patients with IBD remains to be established.

The engineered Vorabody™ V565 is the first domain antibody to be described that has the required potency alongside an adequate intestinal stability profile to support further development for oral administration in IBD. V565 can be reliably produced and purified at high levels from fermentations of *S. cerevisiae* (data not shown), providing the cost efficiency required for an oral biologic. This contrasts with production routes for monoclonal or polyclonal antibodies, which are expensive or unreliable, respectively. VHHs directed against human TNFα have been described elsewhere^32^. The safety and efficacy of V565 in CD are currently being evaluated in a phase II clinical study, with the ultimate aim of providing IBD patients with an option for an orally delivered, more convenient, and safer anti-TNFα therapy.

## Electronic supplementary material

Supplementary Information
