# Supplementary material for: Preclinical Development of a Novel, Orally-Administered Anti-Tumour Necrosis Factor Domain Antibody for the Treatment of Inflammatory Bowel Disease
Source: Sci Rep. 2018 Mar 21;8:4941. doi: 10.1038/s41598-018-23277-7 (PMC5862986; doi:10.1038/s41598-018-23277-7)
Supplement: Supplementary file 1 — Supplementary Information [file 41598_2018_23277_MOESM1_ESM.pdf]

Preclinical Development of a Novel, Orally-Administered Anti-Tumour Necrosis Factor Domain Antibody for the Treatment of Inflammatory Bowel Disease

Authors: J. Scott Crowe[1], Kevin J. Roberts[1], Tim M. Carlton[1], Luana Maggiore[1], Marion F. Cubitt[1], Simon Clare[2], Katherine Harcourt[2], Jill Reckless[3], Thomas T. MacDonald[4], Keith P. Ray[1], Anna Vossenkämper[4] and Michael R. West[1]

1. VHsquared Ltd., 1 Lower Court, Copley Hill, Cambridge Road, Babraham, Cambridge, CB22 3GN, UK
2. Wellcome Sanger Institute, Wellcome Genome Campus, Hinxton, CB10 1SA, UK
3. RxCelerate Ltd. Babraham Research Campus, Cambridge CB22 3AT, UK
4. Blizard Institute, Barts and the London School of Medicine, Queen Mary University of London, London, UK

***Address for Correspondence:** Dr. Scott Crowe, *VHsquared Ltd.*, 1 Lower Court, Copley Hill, Cambridge Road, Babraham, Cambridge, CB22 3GN. Phone: +44 (0) 1223 837650; Email: Scott.Crowe@vhsquared.com

SUPPLEMENTARY DATA

Table S1: Signalling functions of phosphoproteins inhibited following treatment with TNFα-neutralising antibodies in *ex vivo* cultures of inflamed CD and UC mucosal tissue. Inflamed mucosal biopsy tissue from patients with CD or UC was incubated with either V565, IFX or corresponding control antibodies (ID2A, IgG1) and the phosphorylation status of 39 tissue proteins analysed on PathScan arrays as described in the methods section and presented in Figure 4. The phospho-intensity signals obtained for the V565 and IFX treated biopsies were normalised using the corresponding ID2A or IgG1 control intensity values to calculate a "% inhibition" score for each of the 39 proteins. Average "% Inhibition" values for all 39 proteins were then calculated for the n=4 CD or n=4 UC biopsies in each treatment group. The average % Inhibition values are presented for phosphoproteins in V565 (250nM)-treated CD tissue and V565 (300nM) treated UC tissue and infliximab (IFX, 67nM)-treated UC tissue with effects of >70% inhibition highlighted in green. Functions and/or cell types potentially affected by the inhibition of individual RTKs or signalling proteins have been identified.

Phosphoprotein Target	Treatment Effect (% Inhibition)*			Possible Function and Cell Association
	CD	UC	UC	
	V565	V565	IFX	
TrkA	70.6	91.6	77.3	NGF Receptor: Regulatory role in CD and UC
TrkB	66.7	94.9	75.2	NGF Receptor: Regulatory role in inflammatory disease
Met	80.0	98.0	68.9	HGF Receptor TK / Epithelial cell regulation
Ron	80.6	16.0	61.1	Activation of epithelial cells & macrophages
Ret	86.1	100.0	48.2	Receptor tyrosine kinase – cell proliferation
FLT3	80.3	62.8	58.8	Haematopoiesis & immune cell regulation
M-CSFR	86.0	72.9	49.8	Macrophage activation
EphA1	73.1	82.8	ND	Expressed in intestinal epithelium in IBD
EphA2	57.9	84.1	62.2	Expressed in intestinal epithelium in IBD
EphA3	87.1	78.9	60.9	Epithelial cell regulation / crypt architecture
EphB1	86.2	72.1	72.3	Epithelial cell regulation / crypt architecture
EphB3	93.9	62.0	40.9	Epithelial cell regulation / crypt architecture
EphB4	100.0	100.0	100.0	Epithelial cell regulation / crypt architecture
Tyro3	100.0	99.2	86.0	Macrophage apoptotic cell clearance
Axl	100.0	100.0	79.0	Macrophage response receptor
Tie2	92.2	94.4	72.8	Macrophage activation / Angiogenesis
VEGFR2	100.0	100.0	59.1	Endothelial cell regulation of angiogenesis
Akt/Thr308	53.7	100.0	98.1	Cell survival / apoptosis
Akt/Ser473	91.8	100.0	91.9	Cell survival / apoptosis
ERK1/2	94.4	100.0	88.0	Cell activation – multiple cell types and signalling pathways
S6 RP	88.6	96.9	81.8	Cell activation – multiple cell types and signalling pathways
c-Abl	98.2	100.0	100.0	Immune cell regulation
IRS-1	61.8	80.8	44.6	Signalling adapter protein
Zap-70	62.4	71.0	30.2	T cell receptor-associated kinase
Src	65.2	73.0	31.8	Kinase expressed in immune cells
Lck	78.4	71.9	43.5	Lymphocyte specific kinase
Stat1	82.6	75.8	39.1	Transcriptional regulation – multiple cell types

	CD1	CD2	CD3	CD4	CD1	CD2	CD3	CD4
	ID2A	ID2A	ID2A	ID2A	V565	V565	V565	V565
	500nM	500nM	500nM	500nM	250nM	250nM	250nM	250nM
**ErbB1**	1721	1826	1194	1034	1352	1344	875	164
**ErbB2**	1224	1458	882	799	1126	955	656	0
**ErbB3**	1786	1860	1027	891	1268	1377	1168	61
**FGFR1**	1038	1247	556	937	922	833	578	224
**FGFR3**	1251	1417	949	1212	919	768	496	75
**FGFR4**	1250	1422	909	874	982	829	478	64
**InsR**	1114	1188	686	394	852	684	270	0
**IGF-IR**	1156	1285	662	337	899	649	344	0
**TrkA**	553	814	339	602	271	326	53	79
**TrkB**	674	861	347	607	306	483	70	71
**Met**	624	821	280	503	252	325	0	0
**Ron**	838	948	588	530	316	352	17	0
**Ret**	523	544	16	129	98	202	0	0
**ALK**	588	345	142	220	38	90	129	53
**PDGFR**	854	573	423	367	165	250	253	151
**c-kit**	958	788	785	458	220	393	280	125
**FLT3**	697	627	888	515	175	288	68	2
**M-CSFR**	722	713	722	344	35	255	111	0
**EphA1**	757	418	504	187	27	66	208	88
**EphA2**	868	471	674	284	124	218	330	168
**EphA3**	691	519	561	315	0	77	208	0
**EphB1**	584	525	542	233	0	160	121	6
**EphB3**	407	299	196	69	0	73	0	0
**EphB4**	246	158	0	280	0	0	0	0
**Tyro3**	264	262	20	315	0	0	0	0
**Axl**	272	207	4	209	0	0	0	0
**Tie2**	611	396	576	630	0	0	0	196
**VEGFR2**	252	136	2	223	0	0	0	0
**Akt/Thr308**	892	440	1075	1563	520	0	1022	498
**Akt/Ser473**	510	291	254	784	0	0	0	257
**ERK1/2**	1484	311	1535	1269	0	0	0	284
**S6 RP**	960	448	850	1316	28	0	157	319
**c-Abl**	386	166	262	644	0	0	0	47
**IRS-1**	961	803	805	1526	167	37	609	841
**Zap-70**	1288	880	1081	1540	186	88	725	908
**Src**	1424	867	1395	1728	312	0	1068	705
**Lck**	841	413	745	948	0	0	280	463
**Stat1**	941	606	1004	1508	4	0	450	365
**Stat3**	2010	1508	1892	2067	293	109	1433	1176

SUPPLEMENTARY FIGURE S1: Phospho-array data for ID2A (control) and V565-treated CD biopsies

Freshly isolated mucosal biopsies from 4 patients with CD (CD1-CD4) were incubated for 24h with control ID2A (500 nM) or V565 (250 nM) antibodies. Lysates were analysed on PathScan RTK signalling antibody arrays. Phosphoprotein intensities obtained for the 39 proteins on the individual patient biopsy arrays are shown. Conditional formatting was applied in Excel using a graded colour scale, with red showing the highest and green the lowest values in the data set.

	UC1	UC2	UC3	UC4	UC1	UC2	UC3	UC4	UC1	UC2	UC3	UC4	UC1	UC2	UC3	UC4
	ID2A	ID2A	ID2A	ID2A	V565	V565	V565	V565	V565	V565	V565	V565	V565	V565	V565	V565
	300nM	300nM	300nM	300nM	30nM	30nM	30nM	30nM	100nM	100nM	100nM	100nM	300nM	300nM	300nM	300nM
**ErbB1**	2488.5	4365.5	1272.5	820	683.5	2096	3981	934.5	2057.5	2810.5	3371	757	1239	5373	4246	921.5
**ErbB2**	2886	4351	1161.5	529.5	731.5	2272	2963	604	1927	2409.5	2564	590.5	860	4588.5	2222	698
**ErbB3**	3712	4536.5	2677	959.5	1157.5	2394	3437	1300.5	2035	3478.5	4041.5	1048	1637.5	5954	2724.5	972.5
**FGFR1**	2688.5	2732.5	613	742	937.5	1089.5	2783.5	801	983.5	1644.5	1489	465	517.5	2477	2527.5	365.5
**FGFR3**	2888.5	3995	2413	1074.5	1141.5	1791.5	3715	1017	1977.5	1750.5	1748.5	969.5	1008	3156	2320.5	490.5
**FGFR4**	3089	3512	1276.5	758.5	1139.5	1608.5	3331	825	1660.5	1561	1120	519	489	3004.5	1432	718
**InsR**	3285.5	3133	1727	403.5	1533.5	1443	3291	526.5	1364.5	1946	1122	304	425.5	2789.5	890.5	636.5
**IGF-IR**	2960.5	1881.5	3062	492	308.5	108	3664.5	752.5	573	2495	1295	367.5	0	2843	1195.5	721
**TrkA**	2572.5	2820	1062.5	254.5	1731	1136.5	1981	149	1199	0	0	184.5	331	0	221.5	0
**TrkB**	3058	2767	562	200	1548	1295.5	1172.5	209	1686.5	0	0	233.5	621	0	0	0
**Met**	2366.5	2484	376	0	1122	946.5	1067	76	1188.5	0	0	87.5	144.5	0	0	10.5
**Ron**	2958	2471	1696.5	60	1573	1346.5	1713	103.5	1052	0	0	360.5	296	0	0	195.5
**Ret**	1573	209	0	0	0	0	0	28	0	0	0	0	0	0	0	170
**ALK**	1532	2073.5	1106	329	1229	1093.5	1271.5	216	902	657.5	0	217.5	739	0	635.5	81
**PDGFR**	2612.5	3073.5	2481	480	1988.5	1687.5	1480	521	1872	1269.5	16.5	386.5	1083	622	679.5	206
**c-kit**	3615.5	4383.5	4155	478	2783	2658.5	2640.5	622.5	2726	1756	279.5	441	1526.5	1131.5	765	253
**FLT3**	2340.5	2405.5	3156.5	180.5	1252	853	1302.5	419.5	739	865.5	0	307.5	115.5	0	0	259.5
**M-CSFR**	2760	2252	3349.5	343.5	1428.5	677	2033	495	700	850.5	0	498.5	0	526	0	292.5
**EphA1**	2092.5	1448	3155	447.5	1678	1047.5	2779	559	1023.5	586.5	0	792.5	555	0	348	140.5
**EphA2**	2434.5	1872.5	2768.5	505.5	1779	1046.5	1577.5	608	1169	869	0	419.5	662.5	0	187	150
**EphA3**	3126	2988	3133	292	2242	1881	2078.5	528	1766	662.5	0	556.5	769.5	0	0	175
**EphB1**	2596.5	1666	3075	181.5	914.5	515.5	1434	482.5	279.5	392.5	0	303.5	0	0	0	202.5
**EphB3**	2557	1458	2717	100	1314	171	603	387	135	0	0	356.5	0	0	0	152
**EphB4**	1841	742	946	0	1273	603.5	1519.5	0	661	0	0	118.5	0	0	0	0
**Tyro3**	2508	1253	1610	85	1653.5	1196	1518	128	1081.5	0	0	147.5	80	0	0	0
**Axl**	2839	1196	1096.5	0	1524.5	909.5	900	89.5	680.5	0	0	29	0	0	0	0
**Tie2**	3019.5	1997.5	2780.5	362	1810	952.5	2890.5	636	1016.5	0	218.5	184	0	0	0	81.5
**VEGFR2**	2741.5	1337.5	2465.5	0	1467.5	0	1389.5	148.5	499.5	0	0	58.5	0	0	0	0
**Akt/Thr308**	1225	255.5	3621	267.5	1243.5	1263	2363.5	543	768	0	0	563.5	0	0	0	0
**Akt/Ser473**	2040.5	631	1516	84	1407	1088	901	198.5	1202	0	0	98	0	0	0	0
**ERK1/2**	2309.5	1171	2789	138.5	1571.5	1248	920.5	264	1188.5	0	0	285	0	0	0	0
**S6 RP**	2771	1903	2317.5	316.5	1778.5	901	1103	770.5	1470.5	0	0	526	0	0	0	39
**c-Abl**	1450	0	2172	100	225	0	1099.5	169	0	0	0	204.5	0	0	0	0
**IRS-1**	1539	618.5	3048	551	1083	1457	2169	725.5	721	62.5	0	640.5	123	0	1542.5	100.5
**Zap-70**	2331	1467.5	3105	607.5	1418.5	1575.5	1902	845	1460.5	0	0	828	324	400	1576	147
**Src**	2377	1467.5	2802.5	500.5	1576	1010.5	1555	723	1427.5	0	0	505.5	0	806.5	953	95
**Lck**	2308.5	1409	2439	494	1390.5	1035	812	746.5	986.5	0	0	419.5	0	967	412.5	132
**Stat1**	2382	1522.5	2213	463	1130.5	818.5	90	834	667.5	0	0	139	0	820	0	198.5
**Stat3**	2070	292.5	4832.5	1384.5	958.5	0	3311.5	1702	147.5	2060	0	1311.5	0	4395	3098.5	809

SUPPLEMENTARY FIGURE S2: Phospho-array data for ID2A (control) and V565-treated UC biopsies

Freshly isolated mucosal biopsies from 4 patients with UC (UC1-UC4) were incubated for 24h with either ID2A control (300 nM) or V565 (30 nM, 100 nM or 300 nM) antibodies. Lysates were analysed on PathScan RTK signalling antibody arrays. Phosphoprotein intensities obtained for the 39 proteins on the individual patient biopsy arrays are shown. Conditional formatting was applied in Excel using a graded colour scale with red showing the highest and green the lowest values in the data set.

	UC1	UC2	UC3	UC4	UC1	UC2	UC3	UC4
	IgG	IgG	IgG	IgG	IFX	IFX	IFX	IFX
	67nM	67nM	67nM	67nM	67nM	67nM	67nM	67nM
**ErbB1**	3409.5	4345.5	2502.5	1688.5	3014	0	1275.5	742.5
**ErbB2**	3134.5	3487	1765.5	1261.5	2471.5	0	728	329
**ErbB3**	3713.5	4088	2630	1272.5	4057.5	0	973.5	618.5
**FGFR1**	2859	3071	1632.5	1123.5	2192	0	849	795.5
**FGFR3**	3157	3245	2272	1531	2711	0	694	613
**FGFR4**	2816.5	2403	1696	1209	2419	0	680	320
**InsR**	3043	2295.5	1457	958	2877.5	0	514	0
**IGF-IR**	2110	2233.5	1708.5	1076	2561	0	551	0
**TrkA**	2293.5	639.5	734	400.5	1336.5	0	238.5	0
**TrkB**	2436	356	946	505.5	1649.5	0	298.5	0
**Met**	2152	0	600	288.5	1718.5	0	80.5	0
**Ron**	2550.5	0	637.5	272	2600	0	94.5	0
**Ret**	745	0	329	40	1075.5	0	36.5	0
**ALK**	1590	0	1401	696.5	1413.5	0	290	0
**PDGFR**	2307	77.5	1680	697.5	2040.5	547	561	0
**c-kit**	3166.5	549	1817	812.5	3167	536	543	0
**FLT3**	1667.5	0	1317	507.5	1514	0	432.5	0
**M-CSFR**	1748	0	1404.5	496.5	2200.5	0	346	0
**EphA1**	1730	16	1044.5	487	1107	427	356	40.5
**EphA2**	1580	0	1201	469	1302.5	639.5	372.5	0
**EphA3**	1770.5	0	1435.5	440	1699.5	114.5	304.5	0
**EphB1**	1259.5	0	1039.5	338	712	0	275.5	0
**EphB3**	1173	0	805	186.5	1769	0	213	0
**EphB4**	1749.5	0	87	0	0	0	0	0
**Tyro3**	1718	118	402	0	562.5	0	37	0
**Axl**	1358.5	0	336	0	570	0	0	0
**Tie2**	1424	1376.5	606	284.5	852.5	284	170.5	0
**VEGFR2**	1134	0	359.5	0	927	0	0	0
**Akt/Thr308**	1491	521	421.5	246.5	113.5	0	0	0
**Akt/Ser473**	1422.5	719	373.5	20	463.5	0	0	0
**ERK1/2**	1474	2528	299	37.5	710	0	0	0
**S6 RP**	1705.5	386	657	242.5	851	78	18	0
**c-Abl**	290.5	258	332	0	0	0	0	0
**IRS-1**	1915.5	2646	1087.5	610	509	2057.5	341	525
**Zap-70**	2006.5	2454.5	1138.5	724.5	1311.5	2607.5	284.5	598.5
**Src**	1513	2275.5	1201.5	453.5	1205.5	2418	214	313.5
**Lck**	1295	2031	1107.5	468	1003	2621.5	181	15
**Stat1**	1042	1628	854.5	206.5	1128	2071.5	69	0
**Stat3**	1036	4099.5	2564.5	1498.5	963.5	4659	1181	800.5

SUPPLEMENTARY FIGURE S3: Phospho-array data for IgG1 (control) and infliximab-treated UC biopsies. Freshly isolated mucosal biopsies from 4 patients with UC (UC1-UC4) were incubated for 24h with either IgG1 control (67nM) or infliximab (IFX; 30 nM, 100 nM or 300 nM) antibodies. Lysates were analysed on PathScan RTK signalling antibody arrays. Phosphoprotein intensities obtained for the 39 proteins on the individual patient biopsy arrays are shown. Conditional formatting was applied in Excel using a graded colour scale, with red showing the highest and green the lowest values in the data set.

SUPPLEMENTARY METHODS

Kinetic Measurements of Antibody Binding and Dissociation Constants

The binding affinities of V565 and a Fab fragment of adalimumab for human TNFα were determined at 25 °C, using a SwitchSENSE DRX 2400 Instrument (Dynamic Biosensors GmbH, Munich)1. For adalimumab Fab measurements, 200 nM human TNFα-DNA conjugate (Thermo PHC9013) in 10 mM Na_2HPO_4 / NaH_2PO_4 pH 7.4, 40 mM NaCl, 0.05% (v/v) Tween-20 was immobilized on a SwitchSENSE MPC-48-2-Y1 biochip until saturation (no further increase in fluorescence amplitude) was achieved2. Adalimumab Fab solutions at concentrations of 18.5, 56, 167, 500, 1500 pM were flowed over the TNF-DNA loaded biochip at a flow rate of 50 µL/min. Dissociation was recorded for 25,000 s at a flow rate of 10 µl/min. In order to reliably measure the kinetics of V565-TNFα interaction, V565 was chosen as the immobilized ligand to maximize changes in hydrodynamic friction upon complex formation with human TNFα. V565 was covalently attached to 48meric DNA oligomer prior to immobilization on the switchsense biochip surface. Chip surfaces were loaded with 200 nM V565-DNA conjugate in 10 mM Na_2HPO_4 / NaH_2PO_4 pH 7.4, 40 mM NaCl, 0.05% (v/v) Tween-20 to full saturation. Binding experiments were performed using 56, 167, 500, 1500, 4500 pM human TNFα (Thermo PHC9013) in 10 mM Na_2HPO_4 / NaH_2PO_4 pH 7.4, 40 mM NaCl, 0.05% (v/v) Tween-20. Associations were recorded for 600 s at a flow rate of 50 µL/min. The dissociation of V565-TNFα complexes was followed for 30,000 s at a flow rate of 10 µL/min. All measurements were performed in Molecular Dynamics measurement mode, electrically actuating DNA-nanolevers at a frequency of 10 kHz.

V565 Inhibition of Cellular Responses to Soluble and Membrane TNFα:

TNFα-induced L929 cell cytotoxicity: L929 cells (10,000 cells/well) were cultured for 24h in the presence of human or cynomolgus monkey TNFα (500 pg/ml) and actinomycin D (Sigma, A9415, 0.75 µg/mL) together with dilutions of V565 or adalimumab (100µl volume). After 24 h 10 µl of Alamar

Blue (Invitrogen, DAL1100) was added to the cells, and following 2 h incubation 50 µl of 3% SDS was added to each well and OD measured at 590 nm.

NF-kB/SEAP HEK-293 cells and soluble TNFα: NF-kB/SEAP HEK-293 cells (10,000 cells/well) were plated in 50 µl and incubated overnight. Dilutions of V565 and either human or cynomolgus monkey TNFα in culture medium were prepared at 4-fold the assay concentrations and mixed 1:1. The mixtures were subsequently incubated for 1 hour at room temperature before transferring 50 µl of each mix to the plated cells to give final concentrations of V565 standards (0.005 nM to 10 nM) with TNFα (110pM). After 24h incubation, 20 µL of medium was mixed with 200 µL of Quanti Blue medium (InvivoGen). Following 2h incubation at 37°C in the dark with shaking, OD_{620nm} was measured.

NF-kB/SEAP HEK-293 cells and membrane TNFα: NF-kB/SEAP HEK-293 cells (35,000 cells/well) were plated in 50 µl and incubated overnight. V565 dilutions were prepared in HEK medium at 3 fold the assay concentration; then 50 µl of each dilution were added to the cells, and the plates were incubated for ≤ 1 hour. 50 µl of m-TNFα CHO cells (25000 cells/50 µl) prepared in HEK-medium were then added to the HEK-assay plates to give the final V565 assay concentrations (0.1-300nM). After 24h incubation, 20 µL of medium were mixed with 200 µL of Quanti Blue medium. Following 2 h incubation at 37°C in the dark with shaking, OD_{620nm} was measured.

Western Blotting analysis of MMP digest samples: Samples were diluted to the equivalent of 6.6 ng/µL test compound in load dye and 15 µL was loaded (100 ng of each test compound/lane) into a 10% Bis-Tris NuPAGEgel (ThermoFisher, NP0316BOX). The equivalent volume of the 'no test compound' controls was also loaded. Super Signal MW protein Ladder (ThermoFisher, #84785) was added as a standard at 5 µL/lane. Samples were electrophoresed in SDS-MES buffer and transferred to nitrocellulose membranes (ThermoFisher, IB3010) using an iblot (ThermoFisher, 7 minute semi-dry transfer program 3). The membranes were blocked overnight at 4 ^{o}C in block solution (1% BSA, 2 % Marvel, 0.05% Tween20, 1xPBS pH7.4). VHHs ID34F and ID25F were detected using 1) Primary:

pAb 1952 rabbit αQ65B1(-Flag-6xHis) raised at Eurogentec (terminal bleed) at 1/1000 in block solution and 2) Secondary: polyclonal swine anti-rabbit-HRP (DAKO, P0217) at 1/1000 in block solution + 1% normal goat serum (DAKO, X0907). Etanercept and adalimumab were detected using anti-human IgG-γ-chain-HRP (DAKO P0214) at 1/1000 in block solution. Blots were washed for 6x 5 minutes in 25 mL PBST (1xPBS, 0.1% Tween20) between each incubation step to remove non-specifically bound antibody. Blots were developed using SuperSignal™ West Pico PLUS Chemiluminescent Substrate (ThermoFisher #34577) and imaged using ImageQuant LAS4000 (GE) using Chemiluminescensce setting, High Precision, with 10 second exposures.

TNFR2-TNFα Interference ELISA:

Briefly, 1μg/ml etanercept in PBS was coated onto a 96-well Maxisorp ELISA plate overnight at 4°C then incubated with 200 μl block buffer. Dilutions of V565 were mixed with a fixed concentration of TNFα and applied to the plate for 2 hours. TNFα bound to etanercept was detected using P31ABt followed by Extravidin-HRP and TMB substrate (KPL 50-76-00). Reactions were stopped with 0.5 M H_2SO_4 and plates read at 450 nm OD. V565 competition for TNFα binding to etanercept was determined and a standard curve was constructed to interpolate unknown V565 sample concentrations using GraphPad Prism software.

Adalimumab-TNFα Binding Competition ELISA:

ELISA plates were coated with 0.1 μg/ml human TNFα, 250μg/ml BSA in 1xPBS at 4°C overnight, then incubated with 200μl of block buffer. V565 standards and test samples were mixed 1:1 with 800pM biotinylated adalimumab then transferred into the ELISA plates and incubated for 2 hours. After washing, biotinylated adalimumab bound to TNFα was detected by incubation with 1/2000 Extravidin-HRP for 30min. After washing, ELISA plates were developed with TMB substrate and

stopped with 0.5 M H_2SO_4. Plates were read at 450 nm OD. V565 concentrations in the samples were interpolated from a V565 standard curve using GraphPad Prism software.

V565-TNFα binding ELISA

ELISA plates were coated with 100ng/ml TNFα, 5μg/ml BSA in PBS overnight at 4°C. After washing and blocking with 1% BSA in PBS for $\geq$ 1h at RT, plates were ready for use. Sera from mice were initially diluted to not more than 40% serum in 1% BSA, 0.05% Tween 20 in PBS containing NaCl, such that the final NaCl concentration was 0.6M. Pre-dose sera from naïve and DSS colitis mice were pooled separately to provide sufficient sera for analyses. Further serum dilutions were made in 1% BSA, 0.05% Tween 20, 0.6M NaCl in PBS (assay buffer), using a 1.6-fold dilution factor. V565 standards were prepared in the same way, using 40% pooled naïve mouse sera for the highest V565 concentration. Standards and samples were added to ELISA plates and incubated at RT for 2h before, washing and incubating with 1/1000 anti-V565 pAb in assay buffer, for a further 2h. Plates were washed again, then incubated with 1/2000 Swine anti rabbit IgG-HRP for 1.5h. Plates were developed using TMB and after stopping with 0.5 M H_2SO_4 absorbance at 450nm was read in a BMG Fluostar Omega plate reader.

Animal housing conditions:

Mice were given food (LabDiet 50213) and water *ad labitum*. Mice were house in an IVC cage system with aspen wood chip, card tunnel and nesting material as environmental enrichment. All procedures were carried out during the light cycle.

Supplementary References

1. Knezevic J, Langer A, Hampel PA, et al. Quantitation of affinity, avidity, and binding kinetics of protein analytes with a dynamically switchable biosurface. J Am Chem Soc 2012;134:15225–

15228.

2. Langer A, Hampel PA, Kaiser W, et al. Protein analysis by time-resolved measurements with an electro-switchable DNA chip. Nat Commun 2013;4. Available at: http://www.nature.com/doifinder/10.1038/ncomms3099.
